# Supplementary figures and images for: Flooding tolerance of four tropical peatland tree species in a nursery trial
Source: PLoS One. 2022 Apr 6;17(4):e0262375. doi: 10.1371/journal.pone.0262375 (PMC8985972; doi:10.1371/journal.pone.0262375)

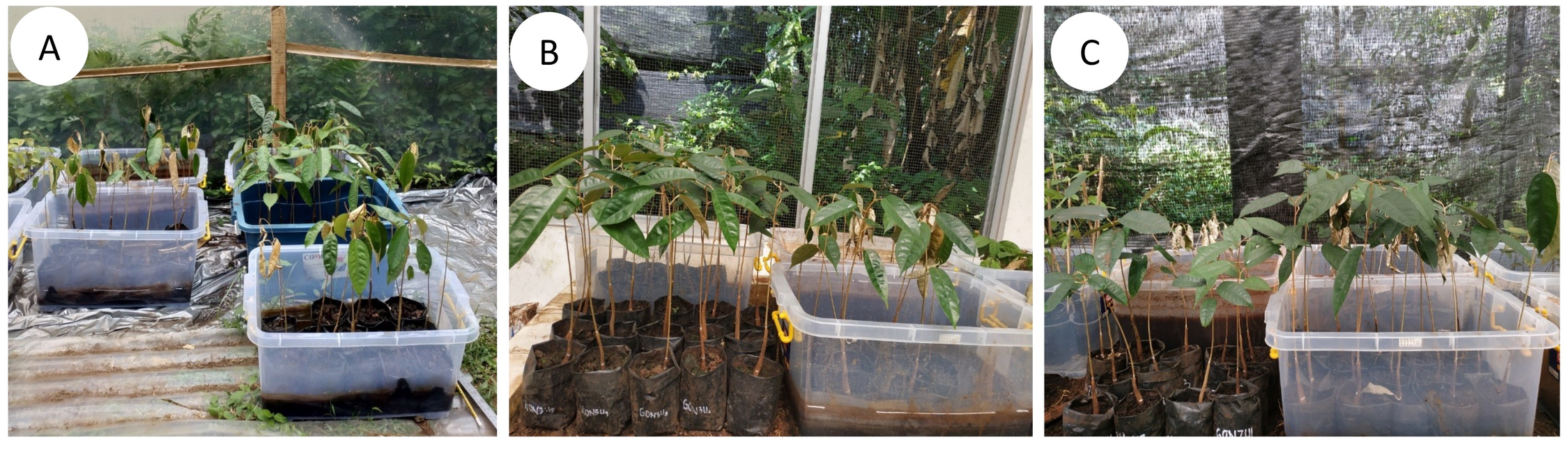

Supplement: S1 Fig — A. Control (0% shading), B. 30% shading, C. 70% shading. (TIF) [file pone.0262375.s001.tif]
