# Supplementary material for: Flooding tolerance of four tropical peatland tree species in a nursery trial
Source: PLoS One. 2022 Apr 6;17(4):e0262375. doi: 10.1371/journal.pone.0262375 (PMC8985972; doi:10.1371/journal.pone.0262375)
Supplement: S1 Table — (PDF) [file pone.0262375.s002.pdf]

Supplementary Information file to

Flooding tolerance of four tropical peatland tree species in a nursery trial

Hesti L. Tata*, Hani S. Nuroniah, Diandra A. Ahsania, Haning Anggunira, Siti N. Hidayati,

Meydina Pratama, Istomo, Rodney A. Chimner, Meine van Noordwijk, Randall Kolka

*Corresponding author email: hl.tata@gmail.com

S1 Table. Plant species used in the study

Plant species	Family
Cratoxylum arborescens (Vahl) Blume	Hypericaceae
Durio zibethinus L.	Bombacaceae
Nephelium lappaceum L.	Sapindaceae
Shorea balangeran Burck.	Dipterocarpaceae
