# Supplementary material for: Flooding tolerance of four tropical peatland tree species in a nursery trial
Source: PLoS One. 2022 Apr 6;17(4):e0262375. doi: 10.1371/journal.pone.0262375 (PMC8985972; doi:10.1371/journal.pone.0262375)
Supplement: S2 Table — (PDF) [file pone.0262375.s003.pdf]

**Supplementary Information file to**

**Flooding tolerance of four tropical peatland tree species in a nursery trial**

Hesti L. Tata*, Hani S. Nuroniah, Diandra A. Ahsania, Haning Anggunira, Siti N. Hidayati,

Meydina Pratama, Istomo, Rodney A. Chimner, Meine van Noordwijk, Randall Kolka

*Corresponding author email: hl.tata@gmail.com

## S2 Table. General Linear Model of Survival Rate

| Source | Type III Sum of Squares | df | Mean Square | F | Sig. |
|---|---|---|---|---|---|
| Corrected Model | 141,564.932[a] | 47 | 3,012.020 | 9.200 | 0.000 |
| Intercept | 4,936,027.696 | 1 | 4,936,027.696 | 15,076.340 | 0.000 |
| Species | 37,600.507 | 3 | 12,533.502 | 38.282 | 0.000 |
| Inundation | 44,306.625 | 3 | 14,768.875 | 45.109 | 0.000 |
| Shading | 1,078.493 | 2 | 539.247 | 1.647 | 0.194 |
| Species * Inundation | 27,976.519 | 9 | 3,108.502 | 9.494 | 0.000 |
| Species * Shading | 12,612.655 | 6 | 2,102.109 | 6.421 | 0.000 |
| Inundation * Shading | 4,582.043 | 6 | 763.674 | 2.333 | 0.031 |
| Species * Inundation * Shading | 13,408.090 | 18 | 744.894 | 2.275 | 0.002 |
| Error | 188,583.702 | 576 | 327.402 | | |
| Total | 5,266,176.330 | 624 | | | |
| Corrected Total | 330,148.634 | 623 | | | |

a. R Squared = 0.429 (Adjusted R Squared = 0.382)
