# Supplementary material for: Flooding tolerance of four tropical peatland tree species in a nursery trial
Source: PLoS One. 2022 Apr 6;17(4):e0262375. doi: 10.1371/journal.pone.0262375 (PMC8985972; doi:10.1371/journal.pone.0262375)
Supplement: S3 Table — (PDF) [file pone.0262375.s004.pdf]

Supplementary Information file to

Flooding tolerance of four tropical peatland tree species in a nursery trial

Hesti L. Tata*, Hani S. Nuroniah, Diandra A. Ahsania, Haning Anggunira, Siti N. Hidayati,

Meydina Pratama, Istomo, Rodney A. Chimner, Meine van Noordwijk, Randall Kolka

*Corresponding author email: hl.tata@gmail.com

S3 Table. Soil chemical properties before treatment

Variable*	value
pH H_2O	3.39
pH KCl	3.18
C-Organic (%)	54.96
N total (%)	0.55
P available (ppm)	19.36
CEC (cmol kg^{-1})	95.97
Pyrit (%)	0.52
Ash content (%)	5.24
Fiber content (%)	0.30

*Method analysis: P-available: Bray & Kurtz (P-Bray I). CEC: NNH_4OAc pH 7.0 extraction. Water content: gravimetri. N-total : Kjedhal.
