# Supplementary material for: Flooding tolerance of four tropical peatland tree species in a nursery trial
Source: PLoS One. 2022 Apr 6;17(4):e0262375. doi: 10.1371/journal.pone.0262375 (PMC8985972; doi:10.1371/journal.pone.0262375)
Supplement: S4 Table — (PDF) [file pone.0262375.s005.pdf]

Supplementary Information file to

Flooding tolerance of four tropical peatland tree species in a nursery trial

Hesti L. Tata*, Hani S. Nuroniah, Diandra A. Ahsania, Haning Anggunira, Siti N. Hidayati,

Meydina Pratama, Istomo, Rodney A. Chimner, Meine van Noordwijk, Randall Kolka

*Corresponding author email: hl.tata@gmail.com

S4 Table. General Linear Model of Relative Growth Rate of Diameter (RGRD)

Source	Type III Sum of Squares	df	Mean Square	F	Sig.
Corrected Model	0.227[a]	47	0.005	17.783	0.000
Intercept	0.651	1	0.651	2394.336	0.000
Species	0.069	3	0.023	84.019	0.000
Inundation	0.008	3	0.003	10.078	0.000
Shading	0.034	2	0.017	61.731	0.000
Species * Inundation	0.014	9	0.002	5.539	0.000
Species * Shading	0.016	6	0.003	9.501	0.000
Inundation * Shading	0.002	6	0.000	1.038	0.400
Species * Inundation * Shading	0.017	18	0.001	3.570	0.000
Error	0.161	591	0.000		
Total	1.586	639			
Corrected Total	0.388	638			

a. R Squared = 0.586 (Adjusted R Squared = 0.553)
