# Supplementary material for: Flooding tolerance of four tropical peatland tree species in a nursery trial
Source: PLoS One. 2022 Apr 6;17(4):e0262375. doi: 10.1371/journal.pone.0262375 (PMC8985972; doi:10.1371/journal.pone.0262375)
Supplement: S5 Table — (PDF) [file pone.0262375.s006.pdf]

Supplementary Information file to

Flooding tolerance of four tropical peatland tree species in a nursery trial

Hesti L. Tata*, Hani S. Nuroniah, Diandra A. Ahsania, Haning Anggunira, Siti N. Hidayati,

Meydina Pratama, Istomo, Rodney A. Chimner, Meine van Noordwijk, Randall Kolka

*Corresponding author email: hl.tata@gmail.com

S5 Table. General Linear Model of Relative Growth Rate of Height (RGRH)

Source	Type III Sum of Squares	df	Mean Square	F	Sig.
Corrected Model	0.043[a]	47	0.001	15.965	0.000
Intercept	0.070	1	0.070	1,239.526	0.000
Species	0.006	3	0.002	36.340	0.000
Inundation	0.006	3	0.002	34.357	0.000
Shading	0.005	2	0.002	41.209	0.000
Species * Inundation	0.008	9	0.001	16.263	0.000
Species * Shading	0.005	6	0.001	14.104	0.000
Inundation * Shading	0.001	6	0.000	2.680	0.014
Species * Inundation * Shading	0.003	18	0.000	3.165	0.000
Error	0.034	592	5.680E-05		
Total	0.204	640			
Corrected Total	0.076	639			

a. R Squared = 0.559 (Adjusted R Squared = 0.524)
