# Supplementary material for: Flooding tolerance of four tropical peatland tree species in a nursery trial
Source: PLoS One. 2022 Apr 6;17(4):e0262375. doi: 10.1371/journal.pone.0262375 (PMC8985972; doi:10.1371/journal.pone.0262375)
Supplement: S6 Table — (PDF) [file pone.0262375.s007.pdf]

Supplementary Information file to

Flooding tolerance of four tropical peatland tree species in a nursery trial

Hesti L. Tata*, Hani S. Nuroniah, Diandra A. Ahsania, Haning Anggunira, Siti N. Hidayati, Meydina Pratama, Istomo, Rodney A. Chimner, Meine van Noordwijk, Randall Kolka

*Corresponding author email: hl.tata@gmail.com

S6 Table. General Linear Model of dry weight (DW) of shoot, root, and root:shoot (RS) ratio

Source	Variable	Type III Sum of Squares	df	Mean Square	F	Sig.
Corrected Model	DW_Shoot	126.812[a]	44	2.882	13.072	0.000
	DW_Root	15.163[b]	44	0.345	6.248	0.000
	RS_Ratio	5.564[c]	47	0.118	10.070	0.000
Intercept	DW_Shoot	142.545	1	142.545	646.501	0.000
	DW_Root	15.121	1	15.121	274.163	0.000
	RS_Ratio	12.777	1	12.777	1,086.910	0.000
Species	DW_Shoot	33.556	3	11.185	50.730	0.000
	DW_Root	1.683	3	0.561	10.174	0.000
	RS_Ratio	1.067	3	0.356	30.260	0.000
Inundation	DW_Shoot	16.042	3	5.347	24.252	0.000
	DW_Root	3.042	3	1.014	18.388	0.000
	RS_Ratio	1.710	3	0.570	48.500	0.000
Shading	DW_Shoot	13.846	2	6.923	31.399	0.000
	DW_Root	2.127	2	1.064	19.284	0.000
	RS_Ratio	0.304	2	0.152	12.939	0.000
Species * Inundation	DW_Shoot	27.134	9	3.015	13.674	0.000
	DW_Root	2.590	9	0.288	5.217	0.000
	RS_Ratio	0.219	9	0.024	2.071	0.040
Species * Shading	DW_Shoot	4.057	6	0.676	3.066	0.009
	DW_Root	0.551	6	0.092	1.665	0.137
	RS_Ratio	1.026	6	0.171	14.546	0.000
Inundation * Shading	DW_Shoot	8.777	6	1.463	6.635	0.000
	DW_Root	2.253	6	0.375	6.807	0.000
	RS_Ratio	0.564	6	0.094	8.002	0.000
Species * Inundation * Shading	DW_Shoot	24.007	15	1.600	7.259	0.000
	DW_Root	2.616	15	0.174	3.162	0.000
	RS_Ratio	0.672	18	0.037	3.178	0.000
Error	DW_Shoot	21.828	99	0.220		
	DW_Root	5.460	99	0.055		
	RS_Ratio	1.129	96	0.012		
Total	DW_Shoot	317.739	144			
	DW_Root	39.247	144			
	RS_Ratio	19.469	144			
Corrected Total	DW_Shoot	148.640	143			
	DW_Root	20.623	143			

	RS_Ratio	6.692	143			

a. R Squared = 0.853 (Adjusted R Squared = 0.788)
b. R Squared = 0.735 (Adjusted R Squared = 0.618)
c. R Squared = 0.796 (Adjusted R Squared = 0.749)
