# Supplementary material for: Flooding tolerance of four tropical peatland tree species in a nursery trial
Source: PLoS One. 2022 Apr 6;17(4):e0262375. doi: 10.1371/journal.pone.0262375 (PMC8985972; doi:10.1371/journal.pone.0262375)
Supplement: S7 Table — (PDF) [file pone.0262375.s008.pdf]

Supplementary Information file to

Flooding tolerance of four tropical peatland tree species in a nursery trial

Hesti L. Tata*, Hani S. Nuroniah, Diandra A. Ahsania, Haning Anggunira, Siti N. Hidayati,

Meydina Pratama, Istomo, Rodney A. Chimner, Meine van Noordwijk, Randall Kolka

*Corresponding author email: hl.tata@gmail.com

S7 Table. Covariance analysis of photosynthesis rate by generating General Linear Model

Source	Type I Sum of Squares	df	Mean Square	F	Sig.
Corrected Model	6,549.715[a]	46	142.385	29.125	0.000
Intercept	19,325.019	1	19,325.019	3,952.902	0.000
Light intensity	5,620.437	1	5,620.437	1,149.652	0.000
Species	172.250	3	57.417	11.744	0.000
Inundation	137.977	3	45.992	9.408	0.000
Shading	352.528	1	352.528	72.109	0.000
Species * Inundation	39.393	9	4.377	0.895	0.532
Species * Shading	91.218	6	15.203	3.110	0.008
Inundation * Shading	48.708	6	8.118	1.661	0.138
Species * Inundation * Shading	87.204	17	5.130	1.049	0.413
Error	532.881	109	4.889		
Total	26,407.615	156			
Corrected Total	7,082.596	155			

a. R Squared = 0.925 (Adjusted R Squared = 0.893)
